# Small-Incision Laparoscopy-Assisted Surgery Under Abdominal Cavity Irrigation in a Porcine Model

**DOI:** 10.1089/lap.2015.0461

**Published:** 2016-02-01

**Authors:** Tatsuo Igarashi, Takuro Ishii, Tomohiko Aoe, Wen-Wei Yu, Yuma Ebihara, Hiroshi Kawahira, Shiro Isono, Yukio Naya

**Affiliations:** ^1^ Center for Frontier Medical Engineering, Chiba University, Chiba, Japan.; ^2^ Department of Anesthesiology, Asahi General Hospital, Asahi, Japan.; ^3^ Department of Gastroenterological Surgery II, Hokkaido University Graduate School of Medicine, Sapporo, Japan.; ^4^ Department of Anesthesiology, Graduate School of Medicine, Chiba University, Chiba, Japan.; ^5^ Department of Urology, Teikyo University Medical Center, Ichihara City, Japan.

## Abstract

***Background:*** Laparoscopic and robot-assisted surgeries are performed under carbon dioxide insufflation. Switching from gas to an isotonic irrigant introduces several benefits and avoids some adverse effects of gas insufflation. We developed an irrigating device and apparatus designed for single-incision laparoscopic surgery and tested its advantages and drawbacks during surgery in a porcine model.

***Materials and Methods:*** Six pigs underwent surgical procedures under general anesthesia. A 30-cm extracorporeal cistern was placed over a 5–6-cm abdominal incision. The abdomen was irrigated with warm saline that was drained via a suction tube placed near the surgical field and continuously recirculated through a closed circuit equipped with a hemodialyzer as a filter. Irrigant samples from two pigs were cultured to check for bacterial and fungal contamination. Body weight was measured before and after surgery in four pigs that had not received treatments affecting hemodynamics or causing diuresis.

***Results:*** One-way flow of irrigant ensured laparoscopic vision by rinsing blood from the surgical field. Through a retroperitoneal approach, cystoprostatectomy was successfully performed in three pigs, nephrectomy in two, renal excision in two, and partial nephrectomy in one, under simultaneous ultrasonographic monitoring. Through a transperitoneal approach, liver excision and hemostasis with a bipolar sealing device were performed in three pigs, and bladder pedicle excision was performed in one pig. Bacterial and fungal contamination of the irrigant was observed on the draining side of the circuit, but the filter captured the contaminants. Body weight increased by a median of 2.1% (range, 1.2–4.4%) of initial weight after 3–5 hours of irrigation.

***Conclusions:*** Surgery under irrigation is feasible and practical when performed via a cistern through a small abdominal incision. This method is advantageous, especially in the enabling of continuous and free-angle ultrasound observation of parenchymal organs. Adverse effects of abdominal irrigation need further assessment before use in humans.

## Introduction

Various modalities of minimally invasive surgery have been developed, sharing the characteristics of small incisions, endoscopic observation, and/or insufflation of carbon dioxide (CO_2_) gas. The expanded field of view of laparoscopy enables meticulous identification of the anatomical structure of organs. However, insufflation of dry CO_2_ gas introduces the risk of gas embolism,^1^ and evaporative cooling causes desiccation and hypothermia,^2^ which can induce postoperative adhesions, problems that can be avoided by humidifying the CO_2_ gas.^3,4^ Furthermore, recent reports indicated that CO_2_ pneumoperitoneum is associated with hepatic injury.^5^ Replacing the CO_2_ gas with an isotonic liquid would avoid desiccation, hypothermia, and excessive abdominal pressure, adding various advantages related to the aquatic properties of irrigants. We previously reported the feasibility of surgical maneuvers under irrigation with an isotonic liquid during cholecystectomy in a porcine model: water-filled laparoendoscopic surgery (WaFLES).^6^

Because the conventional trocars of pure laparoscopic surgery were not ideal for irrigation, we developed a novel irrigation system that works with a small incision and that allows continuous surgical maneuvers with simultaneous observation of laparoscopic and ultrasonographic images. We tested this system during various abdominal and retroperitoneal procedures in a porcine model to confirm visualization of surgical maneuvers and bleeding spots for completing surgery.

## Materials and Methods

Between July 2012 and December 2014, surgery was performed in six specific-pathogen-free pigs with the approval of the local ethics committee for animal experiments. Small-incision laparoscopic surgery was selected to avoid turbulent or jet flow in the irrigant and the mixing of air bubbles in the abdominal cavity, even when replacement of a large volume of irrigant was required.

**FIG. 1.**
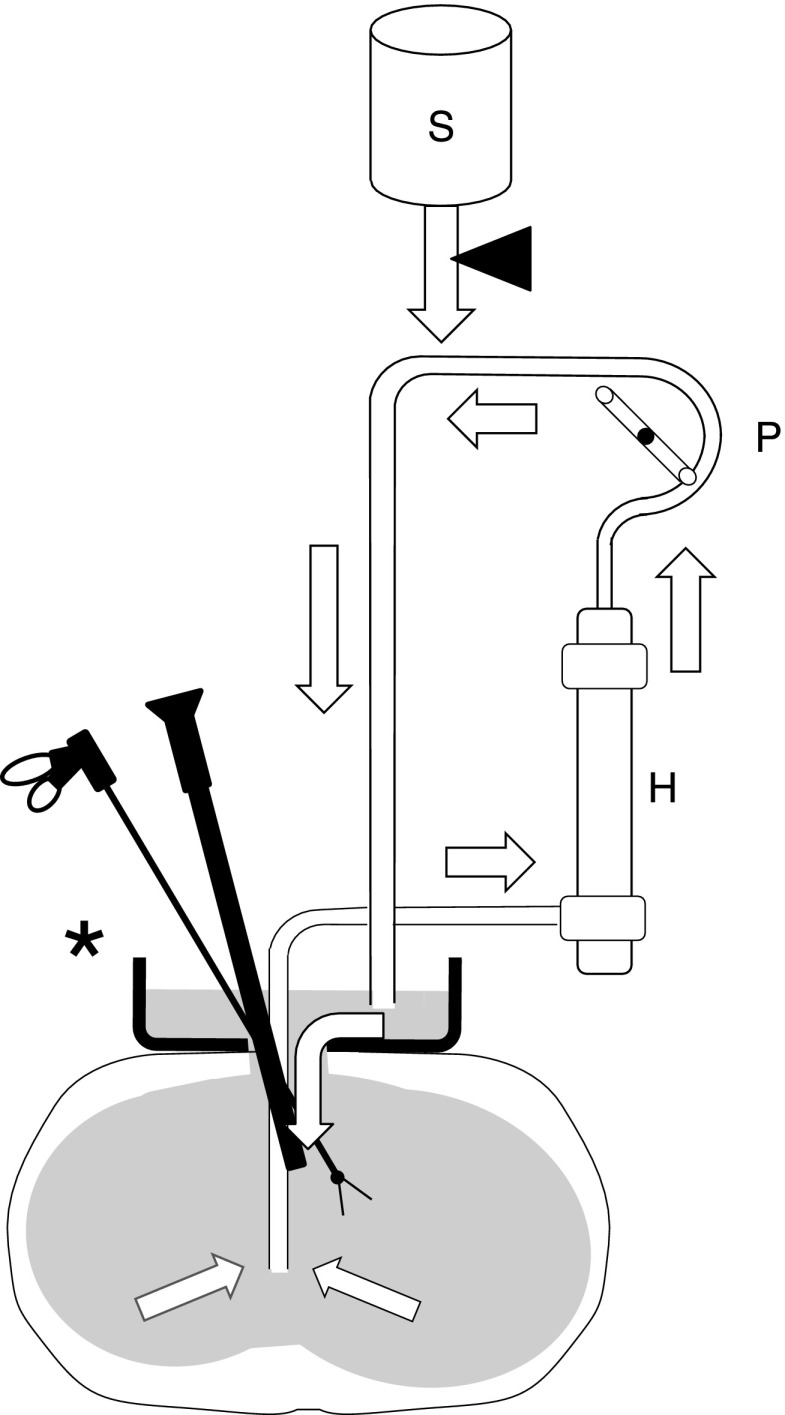
Design of the irrigation system. Arrows indicate irrigant flow direction. The asterisk and closed triangle indicate the extracorporeal cistern and a valve, respectively. H, hemodialyzer; P, pump; S, saline.

**FIG. 2.**
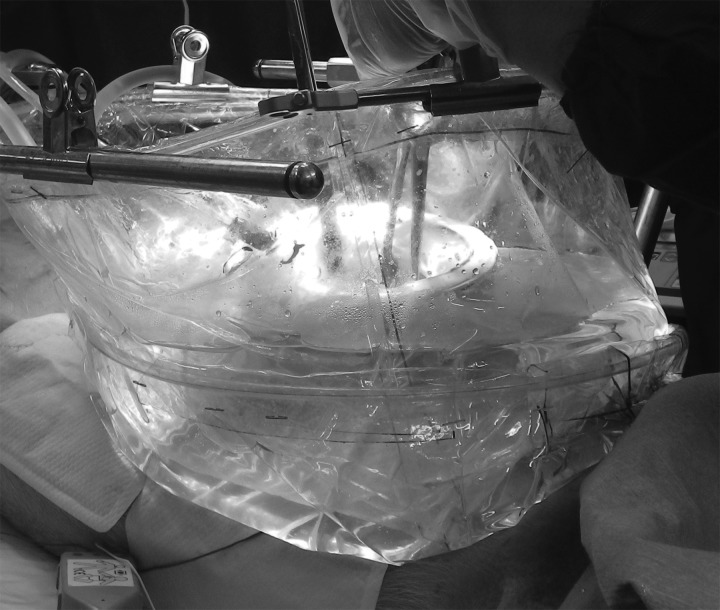
Extracorporeal cistern set over the incision.

**FIG. 3.**
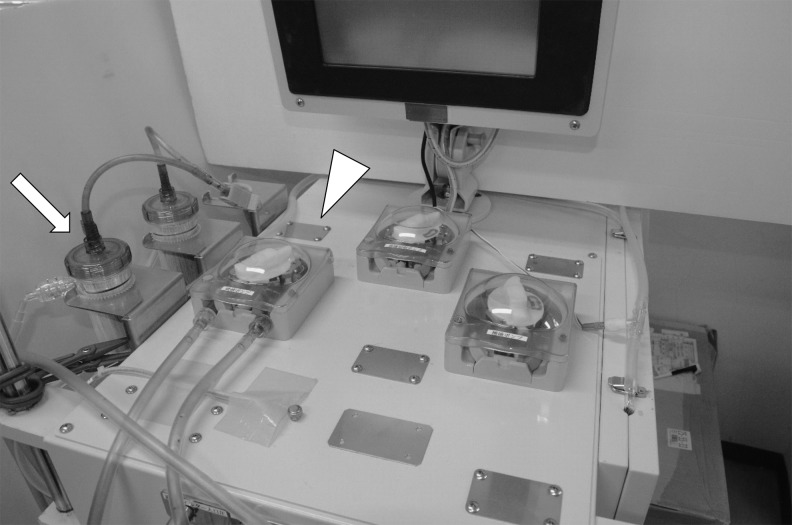
Irrigation pump (triangle) and hemodialyzer (arrow).

Figure 1 shows the surgical system, characterized by a cistern set over the small incision and a closed recirculating circuit. The cistern, 30 cm in diameter and 4–5 cm deep, with a drain at the bottom, was placed over the 5–6-cm abdominal incision (Fig. 2). The recirculating irrigant circuit consisted of high-powered pumps, a heater, and a hollow-fiber dialyzer with a surface area of 2.5 m^2^ (NIKKISO Co. Ltd., Tokyo, Japan) used as a filter (Fig. 3). The pumps were operated at an irrigation speed of 500–1500 mL/minute. Devices and apparatuses used in the study are listed in Table 1. Normal saline was used as the irrigant and was introduced continuously to the abdominal or retroperitoneal cavity via the cistern. The irrigant was collected through a suction cannula set near the surgical site.

**Table 1.** List of Devices and Equipment Prepared for the Surgeries

Irrigation system (self-developed)
• Two pumps and controlling units
• Closed circuit equipped with hemodialyzer
• Thermocontroller unit
Extracorporeal cistern (self-developed)
• Made of polyvinyl chloride and water-resistant adhesive
LAP-PROTECTOR™ (Hakko Co. Ltd., Nagano, Japan)
Abdominal retractor (Thompson Surgical Instruments, Traverse City, MI)
Coagulator
• Bipolar electric cautery (CONMED Corp., Utica, NY)
• Sealing device (ENSEAL^®^, Johnson & Johnson K.K., Tokyo, Japan)
5-mm laparoscope (Karl Storz Endoscopy Japan K.K., Tokyo)
Ultrasonography (Toshiba Medical Systems Corp. [Tochigi, Japan] and Hitachi Aloka Medical, Ltd., Tokyo)

The pigs were prepared under general anesthesia with positive end expiratory pressure (PEEP) set between 5 and 8 cm of H_2_O. The oxygen flow rate was set between 1.5 and 2 L/minute. Liver resection, cystoprostatectomy, nephrectomy, and partial nephrectomy were performed with or without simultaneous ultrasonographic observation. Pigs were placed in the supine position for cystoprostatectomy and liver resection and in a lateral position for nephrectomy and partial nephrectomy.

To allow testing for bacterial and fungal contamination of the irrigant, 13 paired samples were collected under sterile conditions from the hemodialyzer inlet and outlet sites in two pigs. Samples were collected and incubated on soybean-casein digest agar medium at 32°C.

No fluid or drug therapy was administered to the pigs for circulatory support or diuresis. Body weight was measured before and after the procedure as a rough assessment of the volume of irrigant absorbed.

## Results

**FIG. 4.**
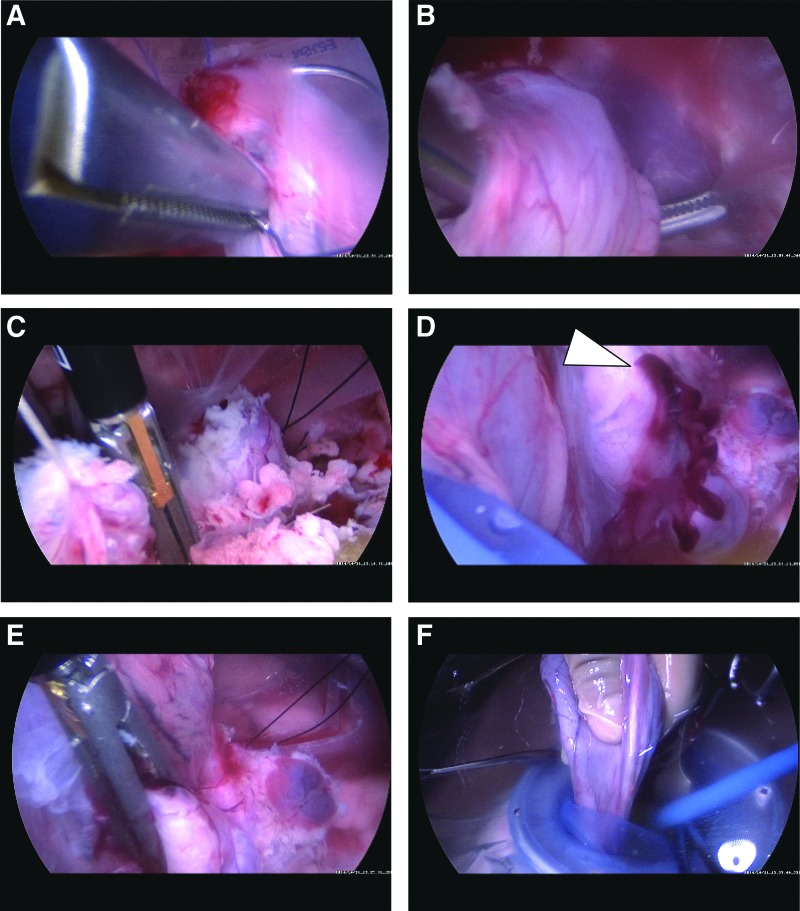
Sequential images of cystoprostatectomy. **(A)** Suture at the dorsal site of the urethra. **(B)** Dissection of the posterior plane of the urethra. **(C)** Transection of the urethra. **(D** and **E)** Venous bleeding from the right lateral wall of the bladder (triangle), controlled with a sealing device. **(F)** Retrieval of the bladder and prostate.

**FIG. 5.**
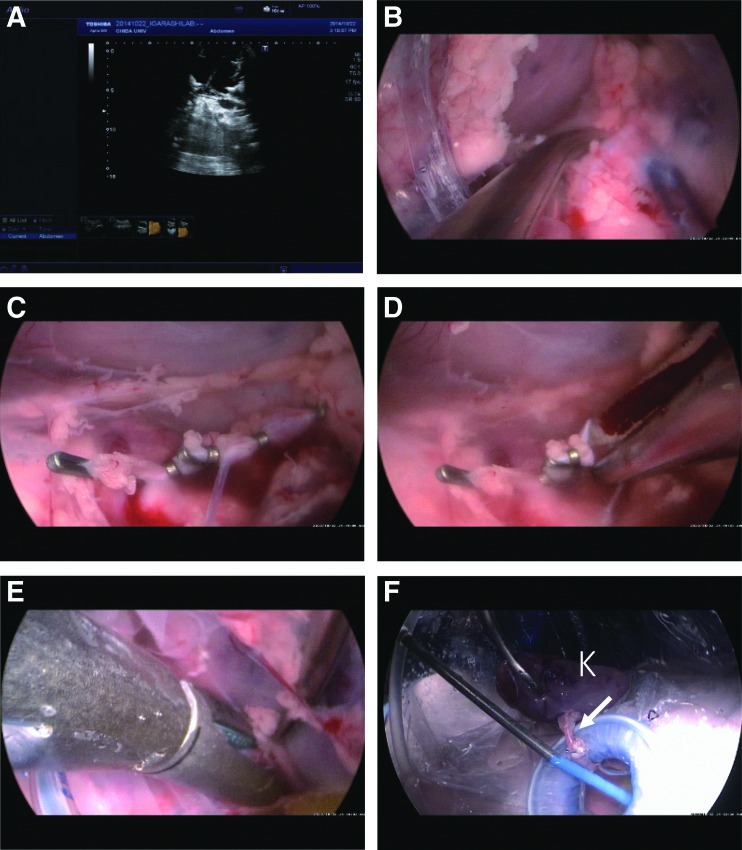
Sequential images of left nephrectomy. **(A)** Ultrasonographic observation. **(B–D)** Dissection, clipping, and transection of the renal artery. **(E)** Transection of the renal vein. **(F)** Retrieval of the kidney (K). The arrow indicates the clipped ureter.

**FIG. 6.**
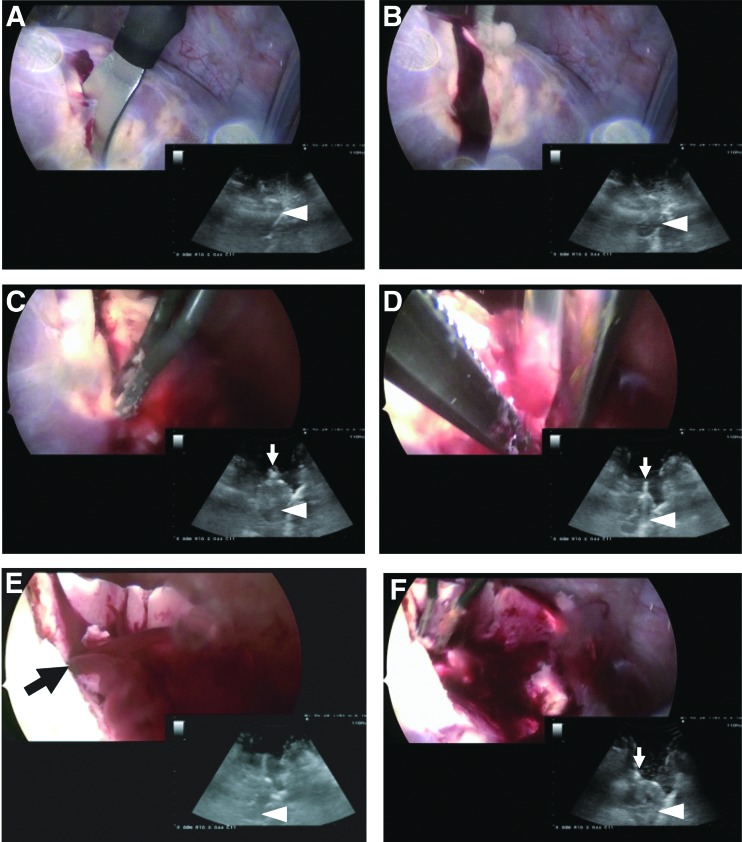
**(A–E)** Sequential images of right partial nephrectomy. White arrows indicate forceps. White triangles indicate the kidney. The closed arrow indicates a bleeding spot in the renal parenchyma.

**FIG. 7.**
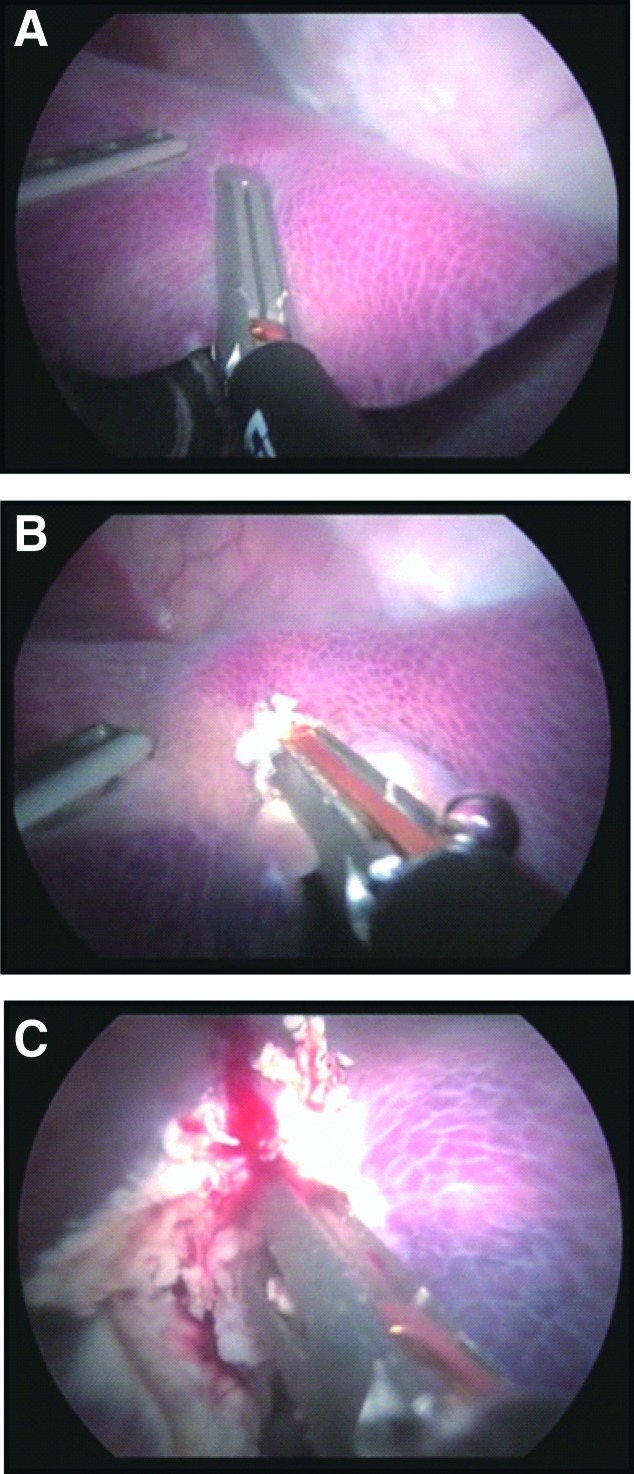
**(A–C)** Sequential images of liver resection.

Skin incision, exploration of the abdominal or retroperitoneal cavity, setting of the external cisterna and suction device, and irrigation apparatus were completed in approximately 10 minutes. One-way flow of irrigant ensured laparoscopic vision by clearing blood from the surgical field, enabling identification of bleeding spots. Through a retroperitoneal approach, cystoprostatectomy was performed in three pigs (Fig. 4), nephrectomy in two (Fig. 5), renal excision in two, and partial nephrectomy in one (Fig. 6). Simultaneous and continuous ultrasonographic assistance was completed displaying a cross-section of the target organ and tip of the device. Through a transperitoneal approach, liver excision and hemostasis with a bipolar sealing device were performed in three pigs (Fig. 7), and excision of the bladder pedicle was performed in one.

The 30-cm cistern did not constrain the surgical maneuvers in every surgery tested; however, transperitoneal nephrectomy could not be performed in all pigs due to interruption of laparoscopic vision and failure of adequate irrigation resulting from floating organs, including the jejunum, ileum, colon, and omentum.

Gram-positive cocci and Gram-negative and -positive bacilli were identified in all samples extracted from the inlet site, with 1–113 colony-forming units/100 mL. A single colony-forming fungal unit was identified in one sample. However, none of the samples from the outlet site showed any colony formation, indicating that bacteria and fungi were trapped by the hemodialyzer.

Body weight was measured in five pigs before and after 3–5 h of saline irrigation. Body weight increased by a median of 0.6 kg (range, 0.4–1.3 kg), which was a median increase of 2.1% (range, 1.2%–4.4%) of initial body weight. The increase in body weight did not depend on irrigation time.

## Discussion

Irrigation or lavage of the abdominal cavity is widely performed in surgical procedures for various therapeutic purposes. Reduction of the risk of surgical-site infection by irrigation/lavage with or without antibiotics has been reported.^7–9^ In cancer control, by reducing peritoneal recurrence and prolonging survival, extensive intraperitoneal lavage is beneficial for patients with resectable advanced gastric cancer.^10,11^ Introducing saline as artificial ascites was reported to be useful during radiofrequency ablation of liver cancer.^12,13^ Because irrigation already had been playing an indispensable role in endoscopic surgery of the “narrow” urinary tract and articular spaces, it seems reasonable that a similar modality could be applied to the wider abdominal cavity, adding the advantages of irrigation/lavage and introducing further options, including a novel image guidance system and separation of components in the drainage. The problems with performing surgery under irrigation in the abdominal or retroperitoneal cavity include dispersion of blood and floating organs that interrupt laparoscopic vision, pathogen contamination of the irrigant, direct introduction of debris into the circulatory system, and possible adverse effects caused by irrigant overload.

In the present study, a novel irrigation/lavage system for the abdominal cavity was developed, and the first problem of blood dispersion in the irrigant was resolved by creating one-way flow around the target organ. In this setting, a small incision plus indirect pouring is adequate as the irrigant inlet, preventing jet flow while allowing introduction of several liters of irrigant per minute. The combination of attaching a cistern over the incision and placing the suction device near the surgical site avoids the turbulent flow and bubbles created when irrigant is introduced from a pump outlet. Thus, the laparoscopic view can be maintained during surgical procedures as long as one-way flow without convection is sustained.

To the best of our knowledge, no article in the English literature has described: abdominal or retroperitoneal surgery with continuous irrigation; investigation of liquid properties, including water pressure, buoyancy, specific heat capacity (much greater than that of CO_2_); or acoustics. Surgical maneuvers in the irrigant differ from those in conventional surgery. Under irrigation, intraabdominal devices are required to handle the floating organs to maintain the view of the surgical field. Membranous bags have been used to wrap the intestines to avoid desiccation during open surgery, and distention balloons have been used to create surgical space in laparoscopic surgery.^14^ Such devices could also be used to keep the surgical field clear under irrigation. Kamiyoshihara et al.^15^ introduced a unique vacuum-packing technique to extract resected tissues from the thoracic cavity. We are tentatively using a similar hollow membranous device to handle organs near the surgical site in the abdominal and retroperitoneal cavities. Nevertheless, floating organs in the peritoneal cavity interrupt laparoscopic vision and suction of irrigant, restricting the surgical coverage of the transperitoneal approach to the retroperitoneal organs.

In transurethral surgery of the lower urinary tract, bleeding vessels are identified and coagulated under irrigation. A similar technique could be achieved in a wider space with the creation of one-way flow and avoidance of convection formation. Under these conditions, the source of bleeding can be identified by following the stream of continuous blood flow, and hemostasis can be achieved by grasping or pinching the bleeding point under direct or laparoscopic vision. Conventional devices, such as sealing devices and bipolar electrodes, worked well in the irrigant. Further development of devices, designed to match the aqueous environment, is warranted.

Direct absorption of debris and/or pathogens into the circulatory system is a serious matter of concern. High fever after transurethral urological surgeries was observed in 11.7% of patients without antibiotic prophylaxis,^16^ and longer operative time was related to sepsis and pulmonary embolism^17^; resolution of these would involve local recovery of debris/pathogens via the suction tube and shorter operative time. Furthermore, the present system is a closed circuit that carries the risk of incubating pathogens. Thus, the system included a dialyzer that can remove bacteria from hemodialysis fluid,^18^ avoiding the development of sepsis by absorbing bacteria that could contaminate and multiply in the irrigant. The present study clearly confirmed that a conventional dialyzer captures bacteria and fungi from the abdominal drainage, indicating that the recycling circuit effectively cleanses the abdominal or retroperitoneal cavity during surgery. However, the closed circuit has a limitation when it comes to managing bowel content spillage when the bowel is opened in any situation. Another emergent circuit for direct drainage and rapid irrigation is now prepared for the present circuit to discard such a contaminated irrigant.

Overload of irrigant in the abdominal cavity provokes adverse effects through excessive intraabdominal water pressure, fluid absorption, and the thermic effects of the irrigant. Critical abdominal compartment syndrome occurs in patients with uncontrolled intraabdominal hypertension over 20 mm Hg, which can be seen with some serious disease states.^19^ Although laparoscopic surgery performed with intraabdominal pressure near 10 mm Hg does not provoke serious circulatory effects to the abdominal organs, injury to the liver is not negligible.^5,20^ Insufflation of CO_2_ exerts uniform pressure on the abdominal organs together with a wide field of view. In contrast, the pressure exerted with irrigation depends on the depth of the water in the cistern, and it is not uniform. In the present study, the water surface in the cistern was set at the body surface level, so that the water pressure at the bottom of the abdominal cavity was below 20 cm of H_2_O (14.6 mm Hg). Organs floating in the upper side of the abdominal cavity are exposed to lower pressure than with CO_2_ insufflation.

In conventional laparoscopic surgery, pneumoperitoneum causes displacement of the diaphragm, which reduces arterial oxygenation. Under such conditions, PEEP improves arterial oxygenation^21^ and reduces cardiac afterload.^22^ Although the distribution of water pressure to the diaphragm differs from that of CO_2_ gas pressure, PEEP set between 5 and 8 cm of H_2_O in WaFLES resulted in arterial oxygenation levels similar to those of conventional CO_2_ pneumoperitoneum (data not shown). Adequate PEEP settings should be investigated further to determine the effects of water pressure and body position.

Pathological manifestations resulting from absorption of large volumes of irrigant during transurethral resection of the prostate have been described. When glycine, mannitol, and/or sorbitol is used as the irrigant, excessive absorption causes hyponatremia, hypervolemia followed by hypovolemia, cardiovascular disturbances, and brain edema.^23^ Hyponatremia can be avoided by using saline as the irrigant at the expense of the unsuitability of using monopolar electrodes. The adverse effects of excessive absorption of saline are primarily volume effects, especially changes in cardiovascular function, edema in critical organs, electrolyte disturbances, and disturbances in serum acid–base balance. Although fluid absorption can be monitored by mixing a trace amount of ethanol in the irrigant,^24,25^ we simply measured body weight in five pigs as a rough estimate of the volume of irrigant absorbed and found that the increase in body weight was less than 4.4% after 3–5 hours of irrigation without any fluid control. The movement and distribution of irrigant and changes in blood gases, serum electrolytes, and acid–base balance should be evaluated further.

Ultrasonography is widely used during surgery to visualize lesions or critical vessels lying deep within organs, including the liver,^26^ prostate and kidney,^27^ and heart,^28^ and in neurosurgery^29^ and gynecological surgery.^30^ These fields benefit from surgical navigation systems that combine laparoscopic vision with computed tomography, magnetic resonance imaging, or ultrasonographic images.^31–35^ However, simultaneous interlacing during surgery is currently not possible because of deformation and dislocation of organs^35^ by the surgical maneuver itself and the effect of gravity. Interposing irrigant between objects and the ultrasonographic probe allows simultaneous real-time ultrasound observation and laparoscopy, with an optimal angle revealing information from within and around the organs. Irrigation would allow the development of a novel navigation system, especially beneficial for resecting parenchymal organs, such as the liver, pancreas, kidney, uterus, and ovary. The present study revealed that simultaneous observation with ultrasonography and laparoscopy was feasible and allowed recognition of the target organ and the tip of the device, indicating that it would fulfill the requirements for navigation.

One drawback of filling the abdominal cavity with irrigant is interruption of the narrow surgical field of view by floating organs. Thus, access via the navel to the liver, gallbladder, kidney, and intrapelvic organs is difficult at present. Thus, the indication for WaFLES would be limited to parenchymal organs in the retroperitoneal and pelvic spaces, as well as the upper abdominal cavity combined with the optimal position and an incision set close to the target organs. It is preferable to place the access port at the costal margin, at a lateral or lower site on the abdomen, or to adopt a retroperitoneal approach. To widen the surgical field, further development of intraabdominal devices is needed. In conventional laparoscopic and robot-assisted surgery of the pelvic organs, the patient is positioned head-down. Irrigant causes the organs to float upward, so that intrapelvic surgery can be performed with the patient in the supine position, and parenchymal organs can be handled with smaller forces than in conventional surgery. Patient positioning, approach, and devices should be redesigned in WaFLES in ways that differ from conventional surgery.

The 5–6-cm incision in the present study is still large and should be minimized. Preparing devices that avoid irrigant flow from reaching the target organ directly could reduce the size of the incision. Trocars, devices for irrigation, and determination of the irrigating velocity and functional volume of the surgical space are now under development (data not shown).

In conclusion, surgery under irrigation is technically feasible and practical in a porcine model and provides advantages related to the properties of the liquid used.
